# Congenital intestinal volvulus with episodes of pain for long period of time: case report 

**Published:** 2021

**Authors:** Hamid Talebzadeh, Shahrzad Andalib, Mohammad Masoud Andalib

**Affiliations:** 1 *Department of Surgery, School of Medicine, Isfahan University of Medical Sciences, Isfahan, Iran *; 2 *School of Medicine, Isfahan University of Medical Sciences, Isfahan, Iran *

**Keywords:** intestinal volvulus, abdominal pain, duodenal obstruction, intestinal obstruction

## Abstract

The incidence of intestinal volvulus as a cause of abdominal pain is rare in adults and normally presents during infancy. Approximately 90% of patients with volvulus are diagnosed within the first year of life, 80% of whom are diagnosed within the first month of life. The current case was a 34-year-old pregnant woman who was admitted to the hospital due to self-limited episodes of epigastric pain from a young age. The patient complained that the pains had recently worsened. Further clinical investigation led us to invasive intervention due to signs of obstruction, and the patient was transferred to the operating room. The case represents a rare incidence of intestinal volvulus in an adult and its complications through pregnancy.

## Introduction

 Malrotation of the mid-gut (intestinal volvulus) rarely presents in adulthood; it mostly presents in the neonatal period (1-3). A diagnosis of intestinal obstruction during pregnancy poses problems, as vomiting that is an important symptom of obstruction can be attributed to hyperemesis gravidarum, and radiological investigation is avoided during pregnancy. 

Herein, we report a 34-year-old pregnant woman presenting in the third trimester of pregnancy with a bowel obstruction due to congenital malrotation of the mid-gut which required invasive intervention. 

## Case report

A 34-year-old woman in the 33rd week of gestation was admitted to the St. Al-Zahra University Hospital in Isfahan for epigastric pain. The patient described 18 years of self-limited episodes of abdominal pain that sometimes improved with defecation or by consuming over-the-counter analgesics. Her pain in this episode was worse and was not completely suppressed by the medications she had used. 

Since the age of 15, the patient had experienced episodes of pain unrelated directly to a specific position or diet that were frequently relieved by different medications prescribed under a physician’s supervision. Eventually, her pain was diagnosed as an abdominal migraine, and no certain medications were prescribed.

On physical examination, the patient had stable vital signs. The abdomen had no tenderness or guarding. She had signs of obstruction, such as no gas passage or defecation but 2 episodes of vomiting in the prior 48 hours. Blood tests showed no leukocytosis (>12000 in five samples) with mild neutrophilia (76% to 92% in five samples). There were no signs of electrolyte imbalances. Hapatobilliary and pancreatic systems were normal in ultrasonographic imaging and laboratory evaluation. Examination by obstetricians showed the fetal status to be stable. 

After surgery and internal medicine consultation, it was planned to evaluate the GI tract by endoscopy and radiographic imaging with the patient’s informed consent. Despite different work-ups during the patient’s first 24 hours of admission, her pain worsened, and there were no abnormal paraclinical signs.

Due to surgical re-evaluation it was planned to rule out gastrointestinal obstruction by computed tomography (CT) scan. CT scan revealed swerving of the superior mesenteric vein (SMV) around the superior mesenteric artery (SMA) and abnormal anatomic position of duodeno-jejunal junction not crossing posterior to the SMA, suggesting mid-gut volvulus (Figure 1-A). There was no evidence of gastric or duodenal distension. Coordination with clinical findings was recommended.

**Figure 1-A:**
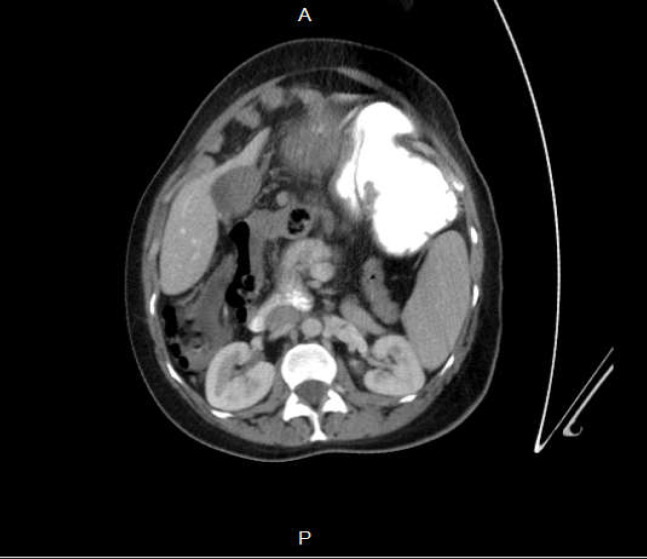
abnormal anatomic position of duodeno-jejunal junction not crossing posterior to SMA

**Figure1-B:**
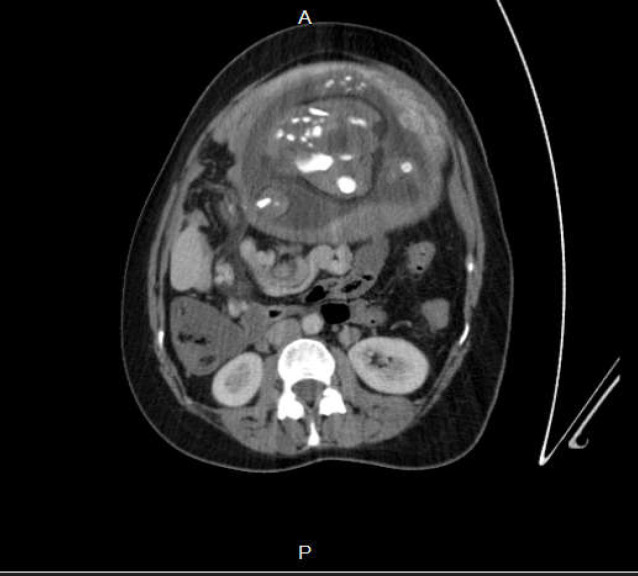
Absence of short bowel and mesothelium in right side of CT represents a term known as mesothelium stalk

In other cut-offs of the CT scan, short bowel was not seen on the right side and no attachment to the retroperitoneum was detected; there was an absence of mesothelium, known as mesothelium stalk (Figure 1-B).

Based on CT scan findings and signs and symptoms of complete obstruction in the previous 72 hours, it was decided to transfer the patient to the operating room to complete evaluations. 

**Figure 2-A:**
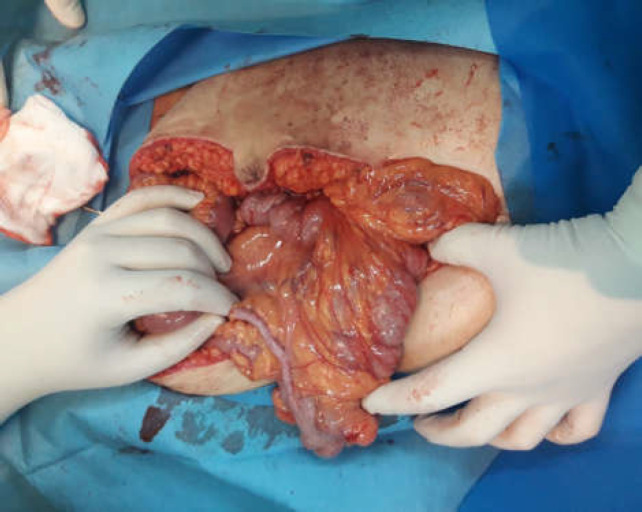
Ascending colon, cecum and appendix were completely free in the cavity, cecum and appendix were detected in LUQ

**Figure2-B:**
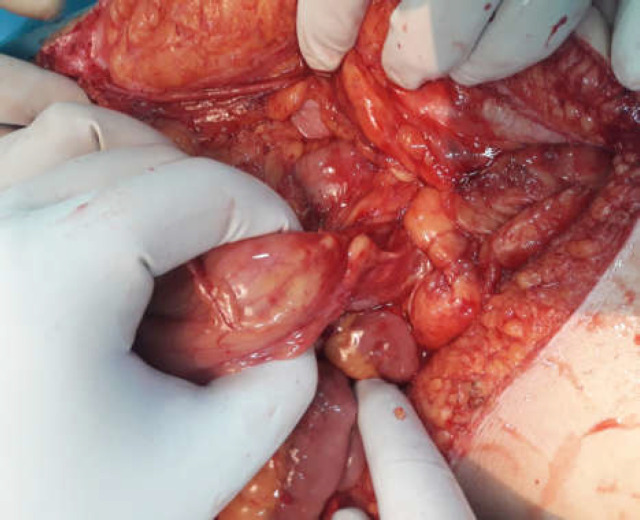
Narrow mesenteric root; one of the main features of this disorder is narrow mesenteric root which causes Intestinal volvulus

After preparation and drape in the presence of a team including general surgeons and gynecologists, with the patient in the supine position, the abdomen was opened with a midline incision. No free fluid, blood, or bowel secretion was seen in the peritoneal cavity. The short bowel was completely collapsed. After exploration, complete volvulus of short bowel was detected and devolvulated immediately. The color of the bowel was appropriate, and peristalsis was normal. Arterial pulse of the mesentery was palpable. The fetus was delivered through cesarean section by the gynecologist and delivered with an Apgar score of 10.

Ascending colon and cecum were completely free in the cavity, and cecum was detected in LUQ (Figure 2-A). Then the general surgeons took action in duodenal exploration with the Kocherisation maneuver and releasing the adhesive bands between the cecum and the right abdominal wall. Widening of the mesenteric roots was done, and dilated and tortuous veins were found all over the root with no bowel wall edema. Finally, an appendectomy was done (Ladd’s procedure). No other pathology was found in the abdomen, and the operation was ended. The patient had a good condition post-operatively and was discharged from the hospital after being able to tolerate an oral regimen with no complications.

## Discussion

Mid-gut malrotation is an uncommon congenital anomaly resulting from a complete nonrotation or an incomplete counterclockwise rotation of the primitive intestinal loop around the superior mesenteric artery during fetal development (2). This malpositioning results in a short stalk of mesentery that easily twists upon itself, resulting in compression of the superior mesenteric artery. This vascular compression results in ischemia of the intestine and necrosis of the intestinal wall in 1–2 h if left untreated (4), and the necrosis can compromise fetal health. Thus, a high level of diagnostic suspicion is needed for an early diagnosis in such a case.

Approximately 90% of patients with malrotation are diagnosed within the first year of life, 80% of whom are diagnosed within the first month of life (5, 6). Intestinal malrotation complicated by mid-gut volvulus, a well-recognized disease entity in infants and children, is rare in adults (3, 6, 7), and its presentation in pregnancy is described only in case reports (8-14). The standard treatment is a Ladd’s procedure, although there are reports of successful detorsion by endoscopy; nevertheless, the risk of recurrence persists. Surgery comprises a counterclockwise detorsion of the bowel, division of anomalous peritoneal fibrous bands (Ladd’s bands), broadening of the mesenteric base, appendectomy, and repositioning of the small bowel and caecum to the right and the large bowel to the left of the abdominal cavity. Anesthesiologists should be aware of these conditions and similar cases to rapidly and definitively control vital signs, replace volume, and correct an electrolyte imbalance during anesthesia and to prepare for resuscitation of the neonate if needed. The knowledge of the management of this condition by anesthesiologists may decrease morbidity and mortality in mothers and neonates.

## Conflict of interests

The authors declare that they have no conflict of interest.
